# Anionic Redox Topochemistry for Materials Design: Chalcogenides and Beyond

**DOI:** 10.1021/acsorginorgau.3c00043

**Published:** 2023-11-07

**Authors:** Shunsuke Sasaki, Simon J. Clarke, Stéphane Jobic, Laurent Cario

**Affiliations:** † Nantes Université, CNRS, Institut des Matériaux de Nantes Jean Rouxel, IMN, F-44000 Nantes, France; ‡ Department of Chemistry, University of Oxford, Inorganic Chemistry Laboratory, South Parks Road, Oxford OX1 3QR, U.K.

**Keywords:** Topochemistry, Anionic redox, Intercalation, Chalcogenides, Low-dimensional materials

## Abstract

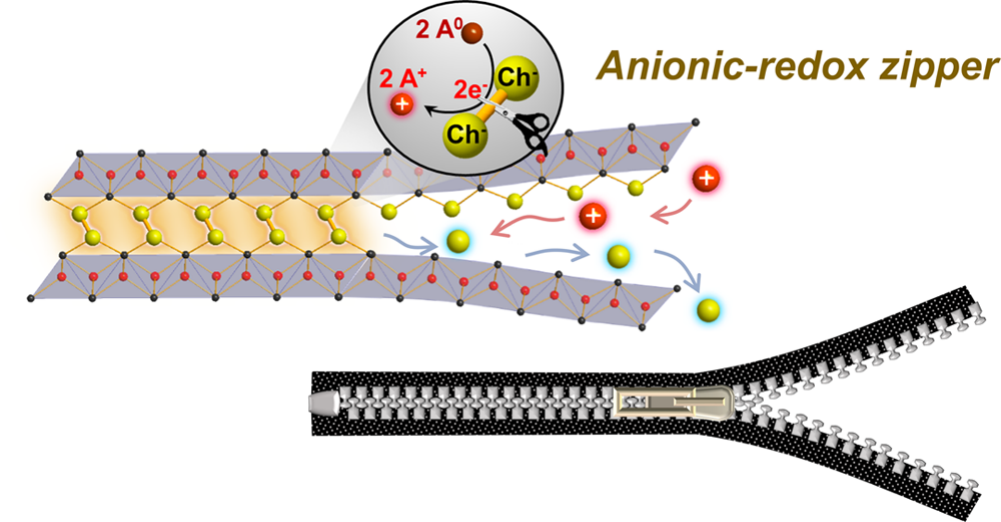

Topochemistry refers to a generic category of solid-state reactions in which precursors and products display strong filiation in their crystal structures. Various low-dimensional materials are subject to this stepwise structure transformation by accommodating guest atoms or molecules in between their 2D slabs or 1D chains loosely bound by van der Waals (vdW) interactions. Those processes are driven by redox reactions between guests and the host framework, where transition metal cations have been widely exploited as the redox center. Topochemistry coupled with this cationic redox not only enables technological applications such as Li-ion secondary batteries but also serves as a powerful tool for structural or electronic fine-tuning of layered transition metal compounds. Over recent years, we have been pursuing materials design beyond this cationic redox topochemistry that was mostly limited to 2D or 1D vdW systems. For this, we proposed new topochemical reactions of non-vdW compounds built of 2D arrays of anionic chalcogen dimers alternating with redox-inert host cationic layers. These chalcogen dimers were found to undergo redox reaction with external metal elements, triggering either (1) insertion of these metals to construct 2D metal chalcogenides or (2) deintercalation of the constituent chalcogen anions. As a whole, this topochemistry works like a “zipper”, where reductive cleavage of anionic chalcogen–chalcogen bonds opens up spaces in non-vdW materials, allowing the formation of novel layered structures. This Perspective briefly summarizes seminal examples of unique structure transformations achieved by anionic redox topochemistry as well as challenges on their syntheses and characterizations.

## Introduction

Functionalities of materials are inherently linked to their underlying structures. As such, chemists aspire to achieve the precise manipulation of constituent atoms and their arrangements. Numerous enzymes in biological systems adeptly cleave or connect targeted chemical bonds, a feat similarly achieved through modern genome-editing technology^1^ and coupling reactions in organic chemistry.^2^ These advancements have enabled tailored designs of intricate molecular structures endowed with the desired functionalities. A similar question then arises concerning inorganic solid-state materials: can a comparable capacity for chemical bond editing be attained for minerals, ceramics, and metals without destroying their overall crystal structures?

**Figure 1:**
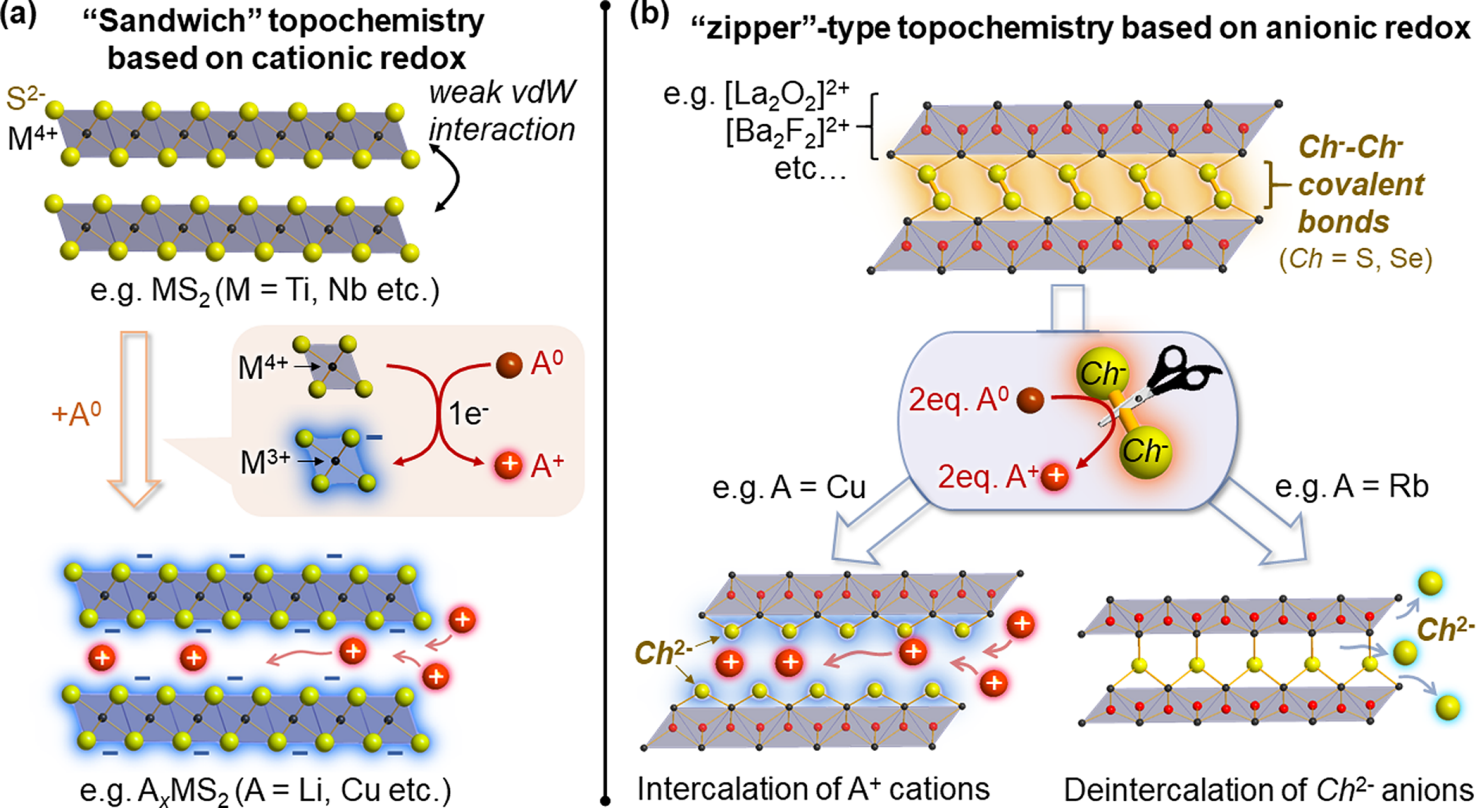
Comparison between (a) conventional topochemistry such as intercalation into 2D van der Waals (vdW) compounds MS_2_, and (b) topochemistry that cleaves chalcogen–chalcogen covalent bonds interconnecting the redox-inert, 2D cationic slabs.

In fact, stepwise structure transformations have been widely examined in extended nonmolecular solids. Already in the 1930s, pioneering studies from Hofmann and several other mineralogists have revealed that layered compounds such as graphite^3,4^ and clay minerals^4,5^ could take up guest species between their rigid 2D slabs. These early investigations were followed by discoveries of many layered transition metal compounds that can host alkali metal cations and organic molecules; some of the seminal examples range from chalcogenides (e.g., MCh_2_; M = Group IV–VI elements, Ch = S, Se)^6,7^ to oxides (e.g., V_2_O_5_ and MoO_3_),^8,9^ halides (e.g., PbI_2_ and BiI_3_),^10^ oxyhalides (e.g., FeOCl),^11^ and phosphorus trichalcogenides MPCh_3_ (M = Fe, Ni, Mn, Ch = S, Se).^12^ Since 2D sheets comprising those host frameworks are bound together only by weak van der Waals (vdW) interactions, intercalation of guest species proceeds under mild conditions, enabling design of metastable sandwichlike compounds inaccessible via conventional high-temperature syntheses (Figure 1a).

Furthermore, these intercalation processes are coupled with a redox reaction between guest species and transition metal cations embedded in the host framework. Taking TiS_2_ as an example, intercalation of elements which form monovalent cations A^+^ accompanied reduction of host Ti^4+^ cations to Ti^3+^ to balance out overall charge neutrality, which was indeed the basis of the first prototypical Li-ion secondary battery conceived by Whittingham and co-workers.^13^ Besides such technological applications, this cationic redox made intercalation processes useful in materials design, particularly for controlling the electronic band filling of low-dimensional compounds. To cite recent remarkable examples, electron doping by intercalation of cationic species induced unconventional superconductivity with e.g. topologically nontrivial states in Cu_*x*_Bi_2_Se_3_ (*x* ∼ 0.3–0.5)^14^ or with Ising spin–orbit coupling in intercalated bulk NbSe_2_.^15^ In the reverse process, deintercalation of K^+^ cations converted semiconducting K_*x*_Fe_1–*y*_S_2_ (*x* ∼ 0.8, *y* ∼ 0.4) into the superconducting 2D FeS,^16,17^ and this was later applied to prepare 2D itinerant ferromagnet CoCh (Ch = S, Se) from KCo_2_Ch_2_.^18^

Intercalations and deintercalations mentioned above can be regarded as a simple host–guest chemistry that inserts or removes chemical species in/from between preformed 2D slabs. Meanwhile, there are increasing numbers of reports that demonstrate drastic structure transformations, yet in stepwise and controlled manner. One highlight example is the use of hydride reagents to remove the apical O^2–^ anions from LaNiO_3_ perovskite, converting this 3D compound into 2D infinite-layer LaNiO_2_ with Ni^+^ under square-planar coordination.^19^ Similar reactions were applied to design 2D SrFeO_2_^20^ and more recently superconducting nickelate Nd_0.8_Sr_0.2_NiO_2_^21^ as well as the related CaCoO_2_ phase.^22^

Such removal of bulky O^2–^ anions transforms the topology of transition metal oxide lattices from 3D to 2D, making accessible unexplored chemical spaces of infinite-layer systems. This exemplifies that the scope of materials that one can conceive will be greatly extended by developing novel types of structure transformation. Such importance of reaction design is well recognized in organic synthetic chemistry, which has been devoting incessant efforts to cut and connect specifically targeted moieties of molecules, including relatively strong C–C^23^ and C–H bonds.^24^ In inorganic solid-state chemistry, stepwise and controlled structure transformations, herein referred to using a generic term “topochemistry”,^25^ have just started to go beyond conventional intercalation chemistry shown in Figure 1a, and its advance will be one of the next major challenges in this field.

To further evolve topochemistry and extend its scope, we have recently proposed to exploit redox activity of covalent anion–anion bonds.^26^ The overall scheme of the concept is depicted in Figure 1b; this novel topochemistry no longer requires the host framework to be a 2D or 1D vdW system with transition metal cations but instead uses non-vdW compounds built of 2D arrays of anionic chalcogen dimers [Ch_2_]^2–^ (Ch = S, Se, Te) interconnecting redox-inert, host cationic slabs. Instead of cationic redox, their anionic [Ch_2_]^2–^ dimers were found to undergo redox reactions with external zerovalent metals, leading to reductive Ch–Ch bond cleavage: [Ch_2_]^2–^ + 2 A^0^ → 2 Ch^2–^ + 2 A^+^. This anionic redox opened up spaces to accommodate the guest A^+^ cations by unfastening covalent Ch–Ch bonds that join the redox-inert 2D slabs. As a whole, this metal intercalation enabled construction of 2D metal chalcogenides [A_2_Ch_2_]^2–^ between host cationic slabs such as [La_2_O_2_]^2+^ and [Ba_2_F_2_]^2+^ layers. While an analogy was often made between conventional intercalation compounds and sandwiches or millefeuilles, this novel topochemistry rather resembles the process that opens or closes a zipper of non-vdW compounds. To highlight the uniqueness of this structure transformation benefiting from anionic redox, we herein call it “zipper”-type topochemistry, taking inspiration from the recent analogy used by Bhaskar et al.^27^

Another way to balance the surplus negative charge imposed by reductive Ch–Ch bond cleavage is to remove part of the anionic constituents. In fact, we discovered that the “zipper”-type anionic redox could also lead to deintercalation of Ch^2–^ anions^28^ (See Figure 1b). Taking La_2_O_2_S_2_ for example, the reaction can be formulated as [La_2_O_2_]^2+^[S_2_]^2–^ + 2 A^0^ → [La_2_O_2_]^2+^[S]^2–^ + A_2_S. This reductive transfer of anionic species resembles electrochemical “conversion” processes well-known for oxides (e.g., CoO + 2Li^+^ + 2e^–^ → Co^0^ + Li_2_O)^29^ and chalcogenides (e.g., FeS_2_ + 4Li^+^ + 4e^–^ → Fe^0^ + 2Li_2_S through Fe–S and/or Li–Fe–S intermediate phases).^30−32^ However, the case in Figure 1b is distinct from those conversion processes since it proceeds in a topochemical manner, i.e., without destroying other structural motifs, and that feature will allow controlled design of new metastable structures.

This short review briefly summarizes several preliminary examples that utilized anionic redox topochemistry for the design of new solid-state materials through unique structure transformations. Then its challenges and perspectives are addressed from a methodological point of view, with the hope of inspiring future advancement in this infant but flourishing field.

## Anion–Cation Redox Competition and Topochemistry

Before describing the actual examples highlighting how anion redox can be useful to alter crystal structures, we herein start from a brief discussion about how anionic species take part in redox processes of topochemistry. We note also that in contrast to its scarcity in materials design, the redox activity of anionic species has been a long-standing subject in electrochemistry.

**Figure 2:**
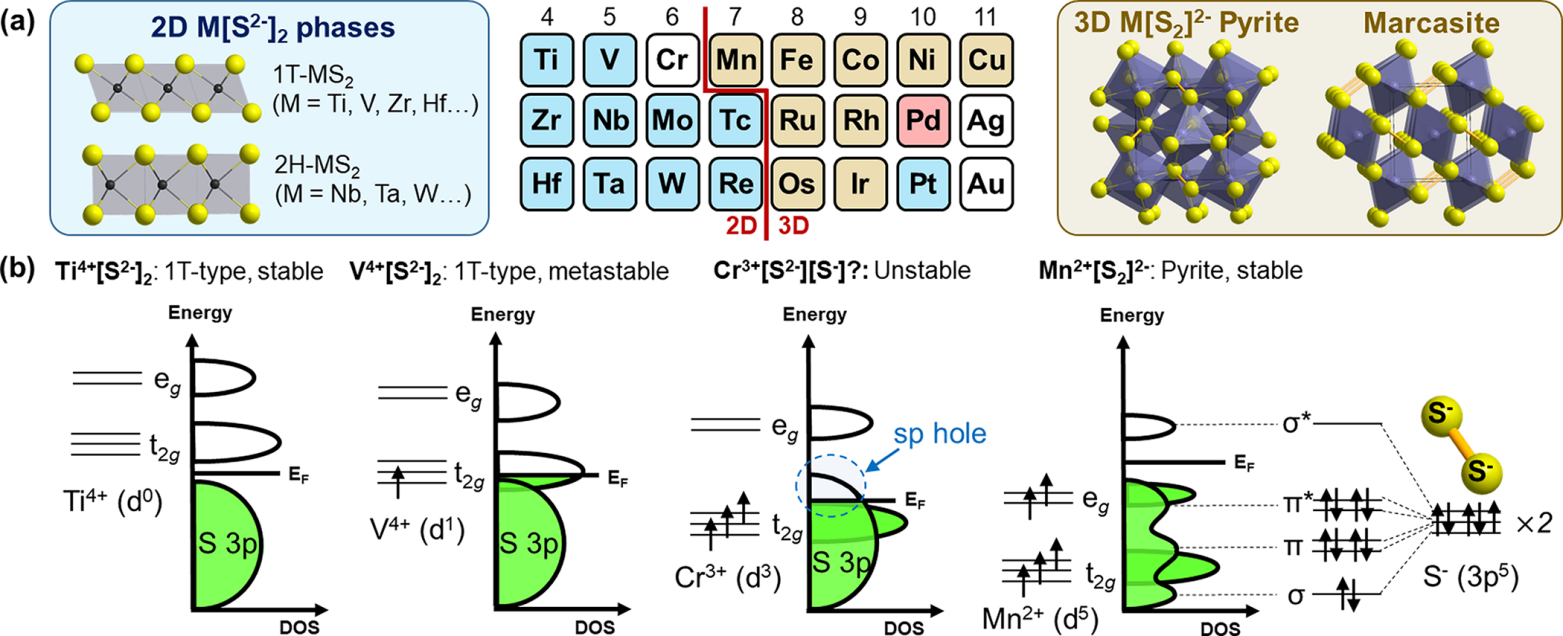
Competition between cationic and anionic redox in transition metal disulfides MS_2_. (a) 2D and 3D structures found among the MS_2_ binary phases. In the periodic table, transition metal elements taking 2D structures are marked in blue while those displaying 3D structures with S–S bonds are marked in brown. PdS_2_, marked in red, shows 2D structure but with S–S bonds.^33^ See refs (34) and (35) for more details including the cases of other chalcogen elements. (b) Schematic diagram describing relative positions of transition metal d bands (*O*_h_ field) and S 3sp bands for M = Ti, V, Cr, and Mn cases. Note that the diagram reflects neither on-site repulsion nor covalency of d-bands in order to represent redox competition in the simplest way. See refs (34) and (36) for the schemes closer to the real band structure.

As described above, it was primarily cationic redox that was used in conventional intercalation chemistry. An archetypical example is the 2D MS_2_ phases with the expected M^4+^[S^2–^]_2_ charge balance (Figure 2a). They adopt different coordination geometries (e.g., octahedral in 1T, trigonal prism in 2H, or both)^35^ depending on the electron count of the transition metal center M, but have in common the presence of a 2D vdW gap and fully reduced S^2–^ anions. Since the transition metal d bands lie well above the filled sulfur 3p bands (Figure 2b), electrons are readily filled or depleted into/from these cationic d bands during intercalation.

On traversing the periodic table from left to right, the transition metal d levels get gradually lowered. In the case of M = V, the 3d t_2*g*_ levels approach the top of the S^2–^ 3p band, hindering depletion of their d-electrons.^36^ VS_2_ with the formal d^1^ configuration and the layered structure cannot be synthesized by reacting elemental vanadium and sulfur at high temperature (V_1+*x*_S_2_ and V_5_S_8_ form instead).^37^ In fact, this metastable compound must be prepared from LiVS_2_ through soft-chemical Li deintercalation.^38^

Such blockage of cationic redox is more important in the M = Cr case (Figure 2b); now the Cr 3d orbitals are lower-lying than the top of S^2–^ 3p band,^36^ so charge balance of the hypothetical CrS_2_ phase requires oxidation of S^2–^ anions. If it were attainable, its likely theoretical formulation would be Cr^3+^[S^2–^][S^–^], with the creation of holes at the top of the S^2–^ 3p band. In reality, such anionic oxidation was not possible even by soft-chemical Li deintercalation from LiCrS_2_. However, DiSalvo et al. reported full delithiation of the alloyed system Li_*x*_Cr_*y*_V_1–*y*_S_2_ (0 ≤ *x* ≤ 1 and 0 ≤ *y* ≤ 0.75),^39^ and Goodenough et al. rationalized activation of anionic redox in this Cr-rich system (e.g., *y* = 0.75) by the “pinning” effect of hybridization between the V 3d band and the top of the S^2–^ 3p band to facilitate creation of anionic sp holes.^36,40^ The same explanation could be applied to the thiospinel CuCr_2_S_4_; its formal charge balance was considered to be [Cu^+^][Cr^3+^]_2_[S^2–^]_3_[S^–^] with holes in the S 3p band that work as redox centers during intercalation of additional Cu,^41^ but those anionic sp holes might actually be hybridized with itinerant Cu^2+/+^ couple.^36^ The concept of “pinning”, i.e., hybridization of cationic and anionic redox couples around a Fermi level, has been applied to design next-generation cathode materials pursuing cumulative electrochemical capacity from both cationic and anionic redox. Fe^2+^ doping greatly improved the electrochemical performance of Li-rich layered sulfide Li_2_TiS_3_^42^ and so does Ni^2+^ doping in the oxide variant Li_2_MnO_3_,^43^ through possible pinning of S^2–/–^ and O^2–/–^ bands. Such hybridization was found effective particularly in Li-rich oxides since it might prevent oxidized O^2–/–^ anions being condensed into gaseous O_2_ molecules.^44^

By going further along the periodic table toward late transition metals (M = Mn, Fe, Co, Ni etc..), anionic sp holes become no longer itinerant and are condensed into covalent chalcogen-chalcogen bonds^34,40^ (Figure 2b). This leads to the occurrence of pyrite- or marcasite-type structures with the expected M^2+^[Ch_2_]^2–^ charge balance, where chalcogen sp bands are split into molecular orbital levels of [Ch_2_]^2–^ dimers. These [Ch_2_]^2–^ dimers should be, in principle, able to accept additional electrons into their antibonding σ* level leading to reductive cleavage of the dimers as in Figure 1b. However, those 3D compounds without a vdW gap tend to undergo alternative nontopochemical structure transformations upon chemical or electrochemical lithiation, giving either Li-M-Ch ternary phases^31^ or conversion products MCh_2–*x*_ + *x* Li_2_Ch.^30,32^ Topochemical syntheses involving explicit cleavage or formation of covalent Ch–Ch bonds are rare, apart from some seminal studies of layered materials.

**Figure 3:**
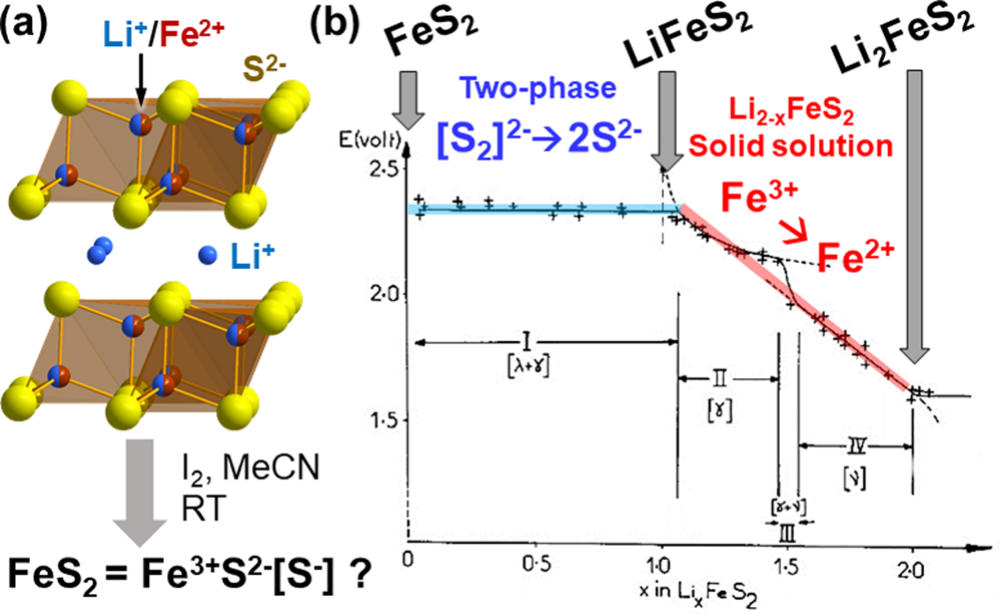
(a) Structure of Li_2_FeS_2_ and its chemical deintercalation. (b) Quasi-equilibrium discharge curve of metastable FeS_2_ prepared by Li deintercalation of Li_2_FeS_2_. Reprinted with permission from ref (45). Copyright (1981) Elsevier.

One such example concerns delithiation of the Li_2_FeS_2_ phase to get a new form of FeS_2_. Dugast et al. reported chemical and electrochemical delithiation of Li_*x*_FeS_2_ down to *x* = 0.14 (Figure 3a).^45^ Mössbauer, EXAFS^46^ and infrared^47^ spectra suggested that this almost Li-free FeS_2_ phase had Fe^3+^ cations in its tetrahedral site, and the possible coexistence of S^2–^ and S^–^ anions. The overall charge balance of this metastable FeS_2_ can be therefore formulated as Fe^3+^S^2–^[[S_2_]^2–^]_1/2_, being distinct from its thermodynamically stable polymorph (i.e., Fe^2+^[S_2_]^2–^ pyrite). This metastable FeS_2_ could be brought back to the original precursor Li_2_FeS_2_ electrochemically,^45−47^ through the two-phase domain converting FeS_2_ to LiFeS_2_ with S–S bond cleavage, followed by Li insertion into Li_2–*x*_FeS_2_ solid solution involving Fe^2+/3+^ cationic redox (Figure 3b).

These results affirmed the topochemical nature of LiFeS_2_ ↔ Fe^3+^S^2–^[[S_2_]^2–^]_1/2_ conversion, and it marked one of the earliest anionic redox topochemical examples that demonstrated its usefulness in the design of new structure types. While it is clearly evidenced that the metastable FeS_2_ has a unique structure distinct from those of pyrite or marcasite, its structure has not yet been experimentally solved yet. Some recent experimental^48^ and theoretical studies^49^ revisited this topochemistry and proposed possible structure models that appear largely plausible (see also the section “Perspectives in Syntheses and Characterizations” later in the article). However, none of these studies reached decisive evidence supporting one proposed structure over the others, leaving room for future comprehensive investigations.

**Figure 4:**
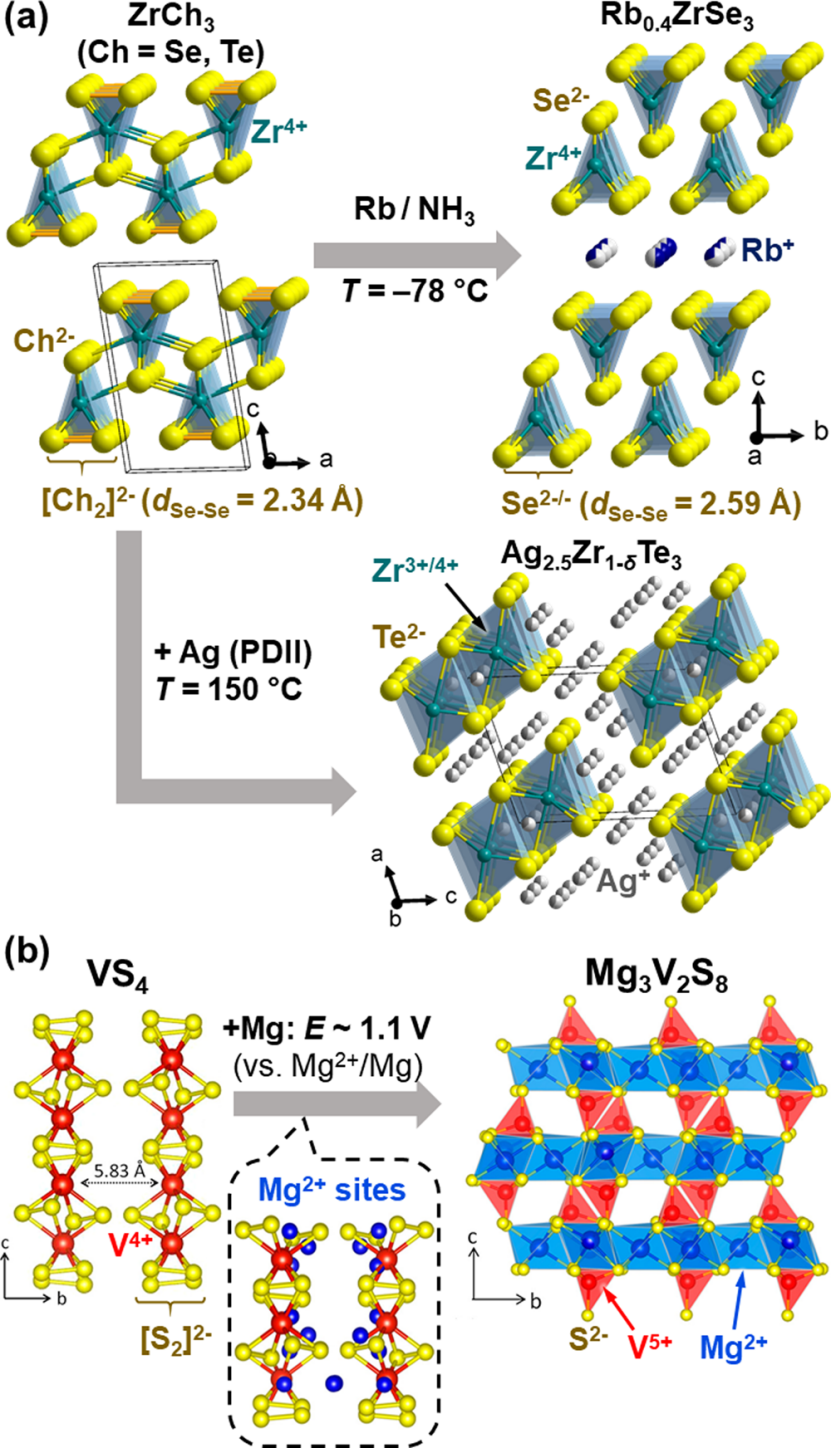
Topochemistry of 2D or 1D vdW polychalcogenides. (a) Intercalation of rubidium^50^ and silver^51^ into ZrCh_3_ (Ch = Se, Te) that gave Rb_0.4_ZrSe_3_ and Ag_2.5_Zr_1−δ_Te_3_, respectively. See Figure 10 for proton-driven ion introduction (PDII). (b) Electrochemical intercalation of Mg^2+^ into VS_4_ giving Mg_3_V_2_S_8_ at *E* ∼ 1.1 V (vs Mg^2+^/Mg). Adapted from ref (52). Copyright (2020) American Chemical Society.

Early transition metals have cationic d bands higher than the top of the anionic sp bands of chalcogen anions, favoring the coexistence of M^4+^ cations and fully reduced Ch^2–^ anions in the MS_2_ phase system (Figure 2b). On the other hand, when further Ch anions are added to form MCh_3_ or MCh_4_ compositions, their anionic sp bands are oxidized into molecular orbital levels of [Ch_2_]^2–^ dimers. In the cases of M = Ti, Zr, and Hf, their transition metal trichalcogenides MCh_3_ present 1D chains of MCh_6_ trigonal prisms extending along *b*-axis of its monoclinic cell (Figure 4a).^53^ These 1D chains are interconnected by a relatively long bond between M^4+^ cation and an apex Ch^2–^ anion while other two anions at the edge form a [Ch_2_]^2–^ dimer, building 2D vdW system with the nominal charge balance M^4+^Ch^2–^[Ch_2_]^2–^. NbCh_3_ and TaCh_3_ (Ch = S, Se) also show similar trigonal prismatic chains, but they are connected in different ways rendering their MCh^2–^[Ch_2_]^2–^ layers more corrugated.^54^

Already in the 1970s, these 2D vdW polychalcogenides have been examined as host materials of chemical^55^ and electrochemical^56^ lithiation. Their reactions with *n*-butyllithium intercalated 3 equiv of Li^+^ cations to give Li_3_MCh_3_ (M = Ti, Zr, Hf, Nb; Ch = S, Se), whose X-ray powder diffraction patterns could be indexed by the original monoclinic cell (See e.g. ZrSe_3_ in Figure 4a) with expansion along *a*- and *c*-axis, but not along *b*-axis indicating the preservation of their 1D chain structure. This discovery was followed by extensive experimental^57,58^ and theoretical investigations^59^ about their structures and reaction mechanisms, as well as application to cathode active materials.^60^ Despite these efforts, the structure model proposed for Li_3_MCh_3_ still remains at the level of a schematic hypothesis inferred from indirect spectroscopic evidence.^58^ In general, intercalation in the MCh_3_ system led not only to poor crystallinity of the products but also to competing side reactions such as conversion processes (e.g., NbSe_3_ + 2*x* Li → NbSe_3-x_ + *x* Li_2_Se),^56,61^ hampering detailed analyses of their structures.

Some significant progress in this topochemistry has been made very recently. In 2022, Elgaml et al. first demonstrated rigorous structure characterizations of soft-chemically intercalated A_*x*_ZrSe_3_ (A = K, Rb, Cs and Ca/NH_3_) phases.^50^ Partial intercalation of alkali metals up to *x* ∼ 0.4–0.5 resulted in elongation of the Se–Se bonds implying reductive cleavage of [Se_2_]^2–^ dimers, as well as longer interchain M^4+^–Se^2–^ distances (Figure 4a). Further extent of metal intercalation was achieved by Fujioka et al.^51^ using their newly developed synthesis method, coined a Proton-Driven Ion Introduction (PDII; see also the section “Perspectives in Syntheses and Characterizations” later in the article). This electrochemical intercalation using solid electrolytes successfully introduced Ag^+^ cations into ZrTe_3_ up to 2.5 equiv, necessarily involving both Te^2–/–^ and Zr^3+/4+^ redox couples (Figure 4a). Their X-ray and electron diffraction studies clearly showcased its gradual transition from 1D trigonal prismatic chains to dimeric octahedral chains through the formation of quasiamorphous phases, where long-range order was lost, except along the 1D chains.

VS_4_ is another variant of early transition metal polychalcogenides, but in this case all sulfur anions are oxidized to [S_2_]^2–^ dimers comprising 1D vdW chains of VS_8_ square antiprisms (Figure 4b).^62^ VS_4_ recently regained attention from electrochemists since its mixture with reduced graphene oxides (rGO) showed excellent performance as a cathode of Li-, Na-, and Mg-ion secondary batteries.^63,64^ Their electrode reactions were then found to go through the ternary Li_3_VS_4_ or Mg_3_V_2_S_8_ phases with V^5+^ and S^2–^ species before reaching their conversion products V^0^ + Li_2_S or MgS.^65,66^ However, the structures of these possible intercalate compounds had remained unsolved due to the poor crystallinity of the products. In 2020, Dey et al. combined computational structure prediction and total scattering analyses to identify the structure of this Mg_3_V_2_S_8_ phase (Figure 4b),^52^ which displayed a strong structural filiation with the parent VS_4_ phase.

The host materials addressed above have vdW gaps in their structures, and thus, their topochemistry may be categorized as a variant of classic intercalation processes (Figure 1a). Nevertheless, it was clear from the recent examples shown in Figure 4 that the reductive cleavage of covalent anion–anion bonds triggered unique structure transformations. Structure characterization was too challenging when those intercalation compounds were first identified, but today it becomes increasingly feasible by employing modern synthetic and characterization tools. This trend is an encouraging sign for future materials designs through anionic redox topochemistry.

## “Zipper”-Type Topochemistry Based on Anionic Redox

### (1) Intercalation of Metal Cations

In 2017, we proposed to make a use of anionic redox topochemistry for design of layered materials.^26^ Anionic redox involves the formation or cleavage of anion–anion bonds. As depicted in Figure 1b, this anionic redox enables a special type of structure transformation when the anion–anion bonds link host cationic layers. Upon the redox reaction with external metal species, those anion–anion linkers are reductively cleaved, opening up a space to accommodate metal intercalants between host cationic layers. This process resembles opening a zipper made of anion–anion bonds, enabling construction of new layered materials from non-vdW polychalcogenides.

**Figure 5:**
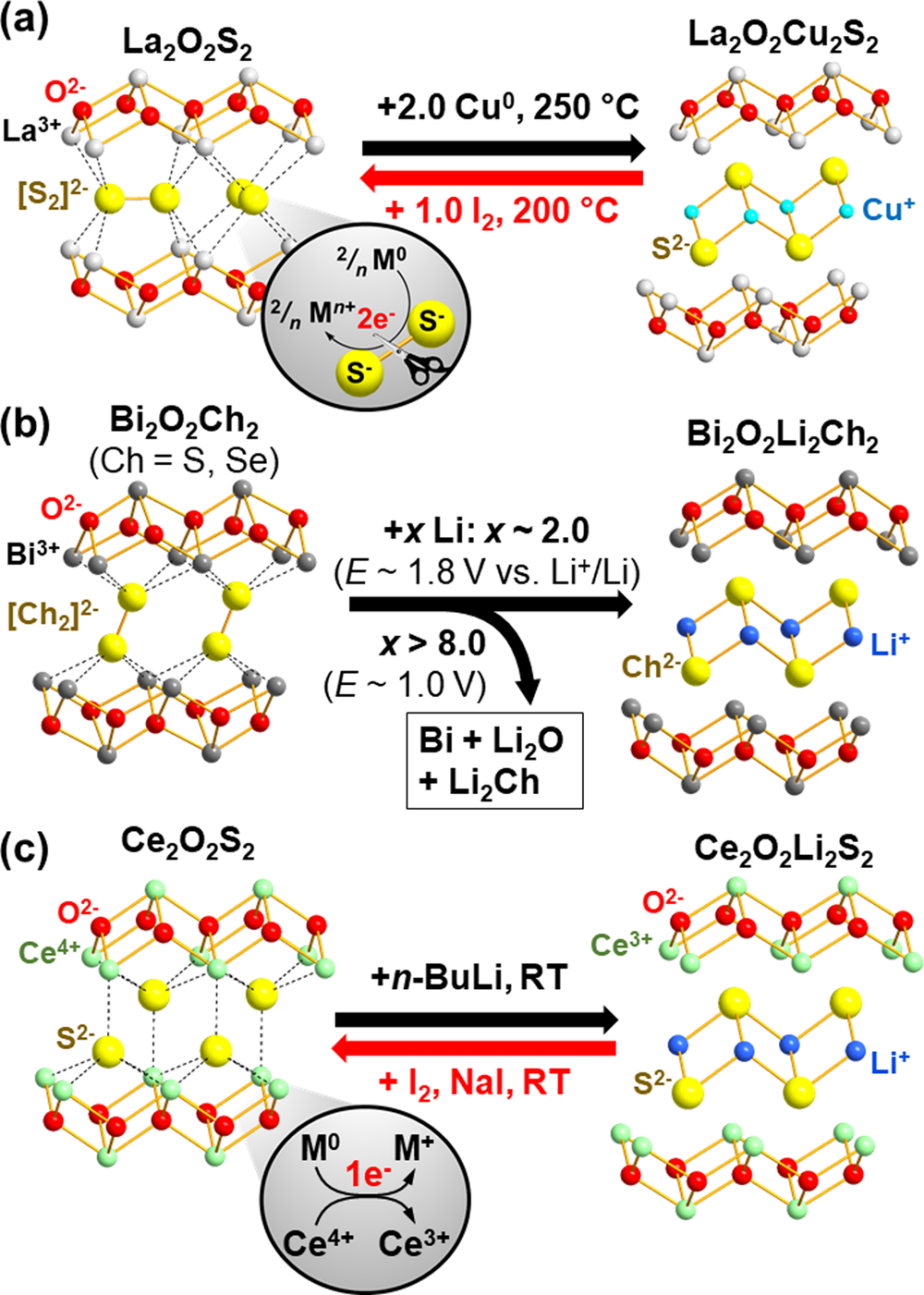
(a) Chemical intercalation of Cu^26^ into [La_2_O_2_]^2+^[S_2_]^2–^ and (b) electrochemical intercalation of Li into [Bi_2_O_2_]^2+^[Ch_2_]^2–^ (Ch = S, Se) that was in competition with nontopochemical conversion.^71^ (c) Chemical lithiation of [Ce_2_O_2_]^4+^[S^2–^]_2_ driven by cationic Ce^3+/4+^ redox.^72^

The proof-of-concept experiments of this novel topochemistry were first performed on La_2_O_2_S_2_ and Ba_2_F_2_S_2_, the compounds where redox-inert, host cationic [La_2_O_2_]^2+^ or [Ba_2_F_2_]^2+^ slabs are interconnected by anionic [S_2_]^2–^ dimers (Figure 5a). Our recent DFT calculation revealed that sulfur 3sp bands in these compounds were split into respective molecular orbital levels of [S_2_]^2–^ dimers^67,68^ in a similar way to the MnS_2_ case shown in Figure 2b, leaving one antibonding σ* orbital available for redox reaction with external reducing agents. Accordingly, if metal intercalants M^0^ are sufficiently reducing to donate their electrons to the σ* level of [S_2_]^2–^ dimers, then these non-vdW host materials should have their S–S bonds broken to accommodate metal intercalants M^*n*+^.

To verify this, La_2_O_2_S_2_ and Ba_2_F_2_S_2_ were heated with Cu^0^ powder in pressed pellets, at low temperature (*T* = 250–275 °C), where conventional precursors without [S_2_]^2–^ dimers could not produce the intercalated quaternary phases (Figure 5a).^26,69^ The reaction swiftly proceeded within a few hours with an intermediate grinding, giving [La_2_O_2_]^2+^[Cu_2_S_2_]^2–^ or [Ba_2_F_2_]^2+^[Cu_2_S_2_]^2–^ phases, where S–S bonds were cleaved and 2D copper sulfide layers were formed. Equally, the successful intercalation of Cu was confirmed under solvothermal (Reagent: CuCl + ethylenediamine producing Cu^0^ through disproportionation, *T* = 200 °C) and mechanochemical (Reagent: Cu^0^ powder, planetary ball milling at 800 rpm, 20 min ×2) conditions.^69^

Furthermore, Cu could be deintercalated upon the reaction with I_2_ at 200 °C, recovering the original polysulfide precursor La_2_O_2_S_2_ by oxidation of the S^2–^ anions.^26^ Such reversibility and mild reaction conditions affirm the topochemical nature of those structure transformations.

This reductive intercalation of Cu was then examined in various host materials,^26,70^ ranging from the simple binary disulfides Ba^2+^[S_2_]^2–^ to its homologue Ba^2+^[S_3_]^2–^ built of sulfur trimers as well as the selenide system [La_2_Se_2_]^2+^[Se_2_]^2–^. In all of these cases, the low-temperature solid–solid reactions with Cu^0^ powder produced copper chalcogenides: BaCu_2_S_2_, BaCu_4_S_3_, and LaCuSe_2_ (= [La_2_Se_2_]^2+^[Cu_2_Se_2_]^2–^).

As for oxidative deintercalation, Chaupatnaik et al. recently reported the removal of Cu^+^ cations from [Bi_2_O_2_]^2+^[Cu_2_Ch_2_]^2–^ (Ch = S, Se), being isostructural with La_2_O_2_Cu_2_S_2_, using I_2_ in acetonitrile solution.^71^ This reaction at room temperature (RT) gave the novel layered polychalcogenides [Bi_2_O_2_]^2+^[Ch_2_]^2–^, which were further confirmed to undergo both chemical and electrochemical intercalation of Li^+^ cations (Figure 5b). At further negative potential, this electrochemical lithiation was followed by nontopochemical conversion process, leading to destruction of their overall layered structure: Bi_2_O_2_Li_2_Ch_2_ + 6 Li^+^ + 6 e^–^ → 2 Bi^0^ + 2 Li_2_O + 2 Li_2_Ch. Such competition between topochemical lithiation and non topochemical conversion was commonly observed in various polychalcogenide systems (e.g., FeCh_2_, NbSe_3_ and VS_4_).^30−32,61,65^

This anionic redox topochemistry resembling a “zipper” may be best contrasted with that of Ce_2_O_2_S_2_, which was recently prepared from Ce_2_O_2_Ag_1.6_S_2_ by Cassidy et al. using an acetonitrile solution of I_2_ and NaI.^72^ Ce_2_O_2_S_2_ contains lanthanide oxide layers similar to those in La_2_O_2_S_2_, but its Ce cations took the oxidation state of +4 with the [Xe] 4f^0^ configuration and its formal charge balance formulated as [Ce_2_O_2_]^4+^[S^2–^]_2_. Accordingly, there were no longer covalent anion–anion bonds that “zipped up” host cationic slabs (Figure 5c). When this compound was subject to chemical and electrochemical lithiation, Ce^3+/4+^ cations played the role of redox center while S^2–^ anions remained redox inert throughout the process.

**Figure 6:**
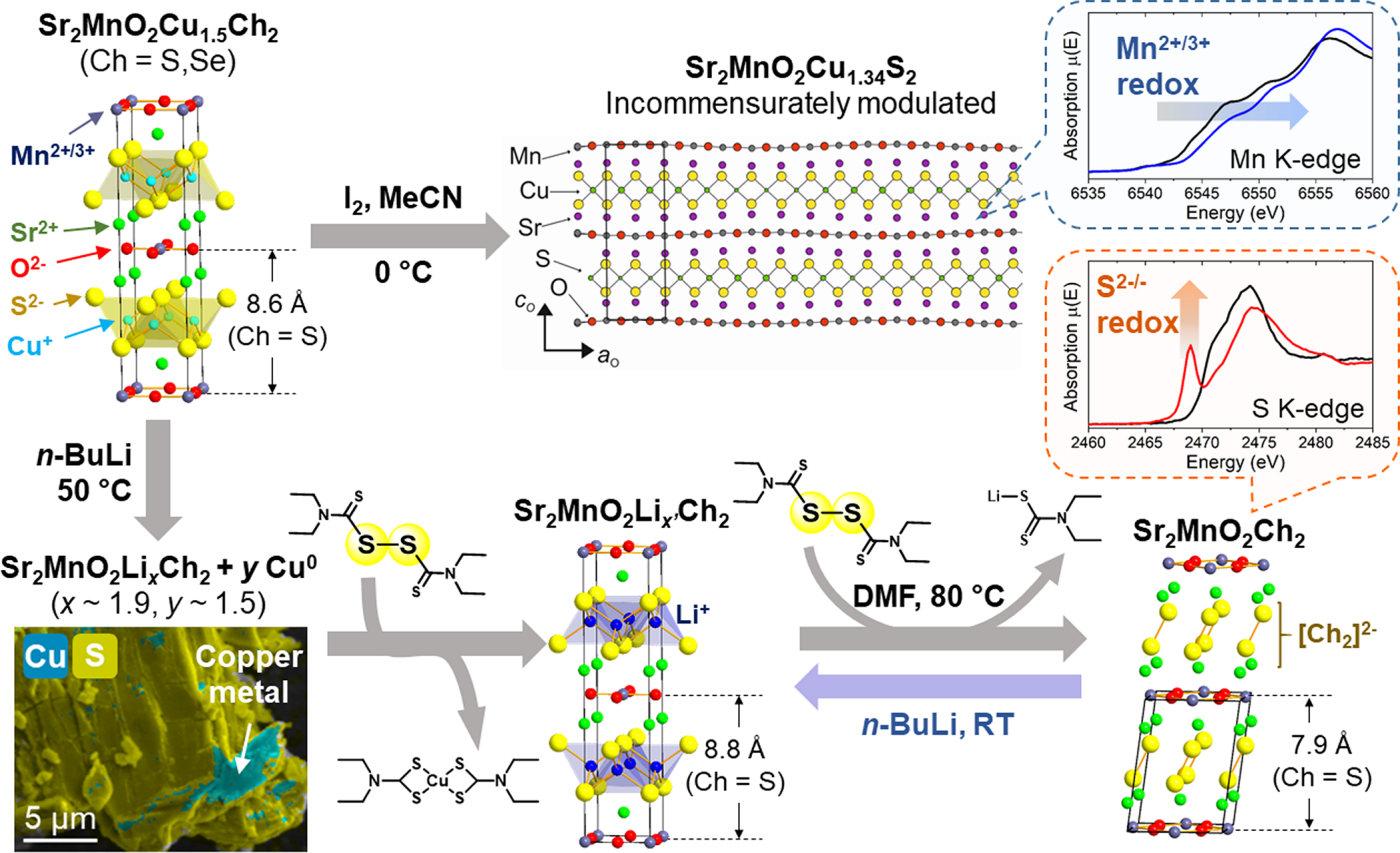
Topochemistry of Sr_2_MnO_2_Cu_1.5_Ch_2_ (Ch = S, Se). (Top) Partial deintercalation of Cu^+^ cations from the Ch = S system using I_2_. The structure of its product Sr_2_MnO_2_Cu_1.34_S_2_ was reprinted with permission under a Creative Commons Attribution 3.0 from ref (73). Copyright (2015) AIP Publishing. Mn K-edge XANES spectra (black: precursor; blue: product) indicated that the partial deintercalation led to oxidation of Mn^2+/3+^ cations. (Bottom) Multistep topochemistry starting from reductive Cu–Li exchange (image: SEM/EDX map evidencing extrusion of the surface Cu^0^ metals), followed by Cu dissolution and subsequent Li^+^ deintercalation at elevated temperature (*T* = 80 °C). S K-edge XANES (black: precursor, red: product) indicated S^2–/–^ oxidation in the final product. The bottom scheme was adapted with permission under a Creative Commons Attribution 4.0 from ref (74). Copyright (2023) Springer Nature.

It is also possible to switch from cationic to anionic redox in the same system. Sr_2_MnO_2_Cu_1.5_Ch_2_ (Ch = S, Se), a layered compound where the perovskite type Sr_2_MnO_2_ slabs are intergrown with the anti-PbO type Cu_1.5_Ch_2_ slabs, is one of such compounds displaying complex redox competition (Figure 6). As shown in Figure 2b, Mn cations take the +2 oxidation state in pure chalcogenides and further oxidation takes place rather at anionic Ch^2–^ 3sp bands.^34^ On the other hand, Mn cations in Sr_2_MnO_2_Cu_1.5_Ch_2_ were found to exhibit the higher oxidation state at +2.5, owing to heteroleptic coordination of their MnO_4_Ch_2_ octahedra.^75^ This higher-lying Mn 3d band was actually subjected to further oxidation during the partial deintercalation of Cu from Sr_2_MnO_2_Cu_1.5_S_2_. Its reaction with I_2_ in acetonitrile solution removed ca. 10% of Cu^+^ cations from the host framework, giving Sr_2_MnO_2_Cu_1.34(1)_S_2_ with an incommensurately modulated structure (Figure 6, top).^73^ X-ray absorption spectra and magnetometry analyses confirmed the oxidation of Mn^2+/3+^ cations, and this cationic redox altered antiferromagnetic ordering within its MnO_2_ square network from CE-type to an alternative pattern of ferromagnetic stripes.

Cu^+^ cations could not be removed beyond 10% from those oxychalcogenides neither at higher temperature than 0 °C nor by using stronger oxidizing agents such as Br_2_ or NO_2_BF_4_ that led to overall decomposition. Such greater extents of deintercalation required the multistep route going through Cu–Li exchange and subsequent dissolution of the surface Cu^0^ metals (Figure 6 bottom).^74^ Those steps gave Sr_2_MnO_2_Li_*x*_Ch_2_ as the synthetic intermediate, which was then subject to Li^+^ deintercalation by using the organic molecules carrying S–S bonds as oxidizing agents. This oxidative deintercalation at *T* = 80 °C activated anionic Ch^2–/–^ redox, to transform the anti-PbO type Li_*x*_Ch_2_ slabs into 2D array of Ch_2_ dimers. X-ray absorption and magnetometry data indicated that Mn^2+/3+^ cations of the final product were oxidized to a lesser extent than Sr_2_MnO_2_Cu_1.34_S_2_ even though a much larger amount of the monovalent metal in the chalcogenide layer had been removed. In contrast, oxidation of its Ch^2–^ anions was clearly evidenced by the pre-edge feature of their X-ray absorption near-edge structure (XANES) as well as Raman peaks corresponding to Ch–Ch stretching modes.

These contrasting results arising from different routes suggested tight competition between cationic Mn^2+/3+^ redox and anionic Ch^2–/–^ redox. As long as the oxidation state of Mn^2+/3+^ cations does not deviate far from its original value +2.5, (de)intercalation changes the electron count of Mn 3d bands. However, once a greater extent of deintercalation imposes further oxidation, it triggers Ch^2–/–^ redox, keeping the oxidation state of Mn at around +2.5. Similar redox competition was observed also during electrochemical Cu–Li exchange of Sr_2_MnO_2_Cu_3.5_S_3_, a structural homologue of Sr_2_MnO_2_Cu_1.5_Ch_2_ with the thicker copper sulfide layers.^76^ Those observation may be related to redox competition during Mg^2+^ intercalation into VS_4_ (See Figure 4b),^52,64^ where reduction of its [S_2_]^2–^ dimers coincided with oxidation of its V^4+^ cations to V^5+^. To unlock the full picture of this complex redox behavior, it is necessary to carry out further in-depth analyses about how their electronic structures evolve during (de)intercalation processes.

The first preliminary successes in this “zipper”-type topochemistry have been limited mainly to intercalation of monovalent cations (e.g., Li^+^ and Cu^+^). It would be then the next major challenge to extend the scope of intercalants to divalent or trivalent cations, especially to those with open-shell configurations at the d-levels since their interaction will construct 2D transition metal chalcogenides with possible quantum functionalities (Figure 1b).

**Figure 7:**
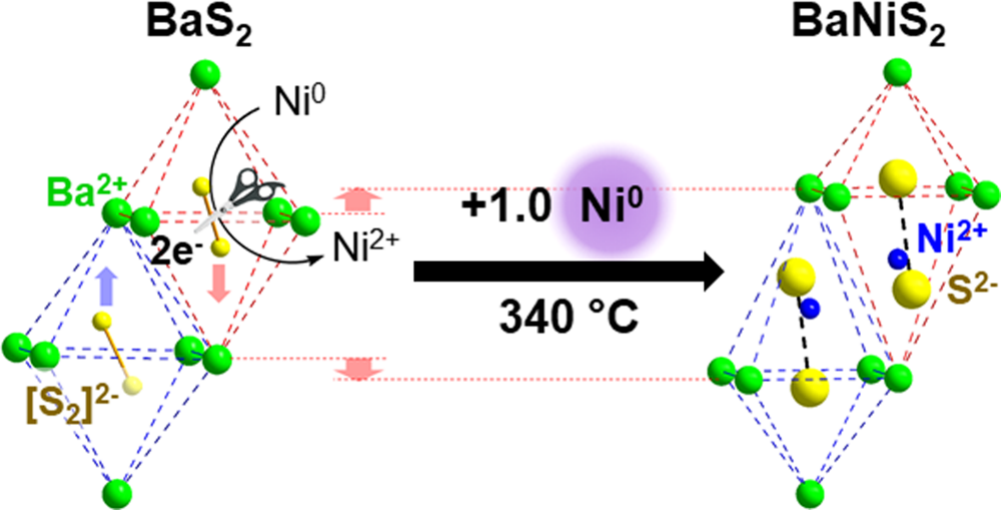
Topochemical structure transformation proposed for low-temperature Ni insertion into Ba^2+^[S_2_]^2–^ giving the quasi-layered Ba^2+^[NiS_2_]^2–^ phase. Reprinted with permission from ref (70). Copyright (2019) Royal Society of Chemistry.

Some proof-of-concept experiments have been made for this using the simple binary polysulfides.^70^ As shown in Figure 7, the structure of Ba^2+^[S_2_]^2–^ has a strong filiation with BaNiS_2_, the quasi-layered compound built of NiS_5_ square pyramids. Accordingly, one can expect that the reductive cleavage of its S–S bonds (i.e., Ni^0^ + [S_2_]^2–^ → Ni^2+^ + 2S^2–^) triggers insertion of these Ni^2+^ cations to construct this ternary phase known as the promising spintronic material,^77^ with a low kinetic barrier. In practice, the solid–solid reaction between BaS_2_ and Ni^0^ powders was found to proceed at *T* = 340 °C, a temperature much lower than the conventional route starting from BaS, Ni and elemental sulfur (*T* = 800–950 °C).^70^ However, this insertion process was in competition with the nontopochemical conversion process BaS_2_ + Ni^0^ → BaS + NiS, which had to be suppressed by delicate control of thermal treatments and the use of BaS_3_ instead of BaS_2_. The similar low-temperature reaction was observed also for BaS_3_ and Fe^0^ giving BaFe_2_S_3_, which was known as the first spin-ladder iron-based superconductor,^78^ but it was also in the competition with the decomposition into other nontopochemical products.^70^ These preliminary results have demonstrated the feasibility of constructing low-dimensional quantum materials through the “zipper”-type topochemistry, but at the same time raised the challenge to steer the selectivity between several competing processes.

The examples described thus far have focused primarily on chalcogenides. On the other hand, secondary battery research has been more focused on the redox of oxygen anions.^44^ In the 2000s, Li_2_MnO_3_ doped with Ni and Co, referred to as Li-rich NMC, was found to be an excellent cathode material for a Li-ion secondary battery,^79^ with extraordinary capacity arising not only from cationic redox of its transition metals and but also from anionic redox of its oxygen. This discovery prompted electrochemists to study the redox activity of oxygen in Li-rich oxides. To date, topochemistry involving the formation or cleavage of an O–O bond still remains elusive. Nevertheless, recent microscopic^80^ and neutron diffraction^81^ studies spotted formation of O–O dimers during oxidative Li^+^ deintercalation of the Li-rich cathode Li_2_IrO_3_. This encourages future attempts to design new compounds through the reversible formation of anionic O–O bonds.

**Figure 8:**
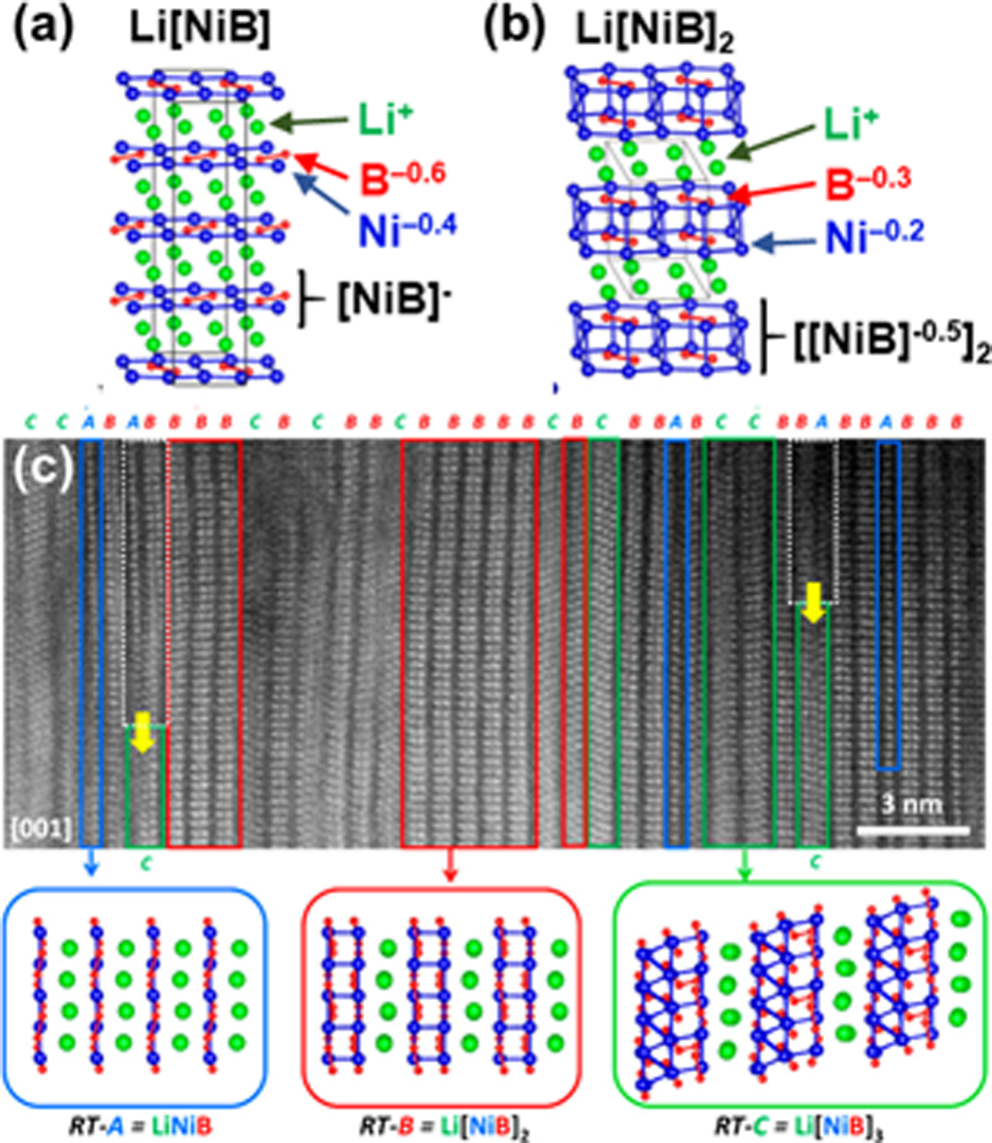
(a) Room-temperature (RT) polymorph of LiNiB and (b) its deintercalated phase Li[NiB]_2_. Net charges were estimated by Bader analysis. (c) HAADF-STEM image of the product after deintercation, highlighting the coexistence of the pristine LiNiB slabs (RT-A), the deintercalated Li[NiB]_2_ double layers (RT-B) and Li[NiB]_3_ triple layers (RT-C). Reprinted from ref (27). Copyright (2021) American Chemical Society.

Dimerization, oligomerization and polymerization of anionic networks are not limited to group 16 elements, but also known among various semimetals and intermetallic compounds built of pnictogens, carbon group and transition metal elements.^82^ Actually, the analogy of “zip-lock” was first used by Bhaskar et al.^27^ to refer to topochemistry of LiNiB that involved Ni–Ni bond formation (Figure 8). They discovered that deintercalation of Li^+^ cations oxidized its anionic [NiB]^−^ single layers, leading to its condensation into double [NiB]_2_ or triple [NiB]_3_ layers that “zipped up” the spaces between NiB slabs through Ni–Ni bond formations. HAADF-STEM image of the deintercalated product (Figure 8c) highlighted the structural diversity of these zipped-up [NiB]_*n*_ (*n* = 2, 3) layers, heralding future prospects to design various 2D metal borides coined MBenes.

### (2) Deintercalation of Sulfur Anions

**Figure 9:**
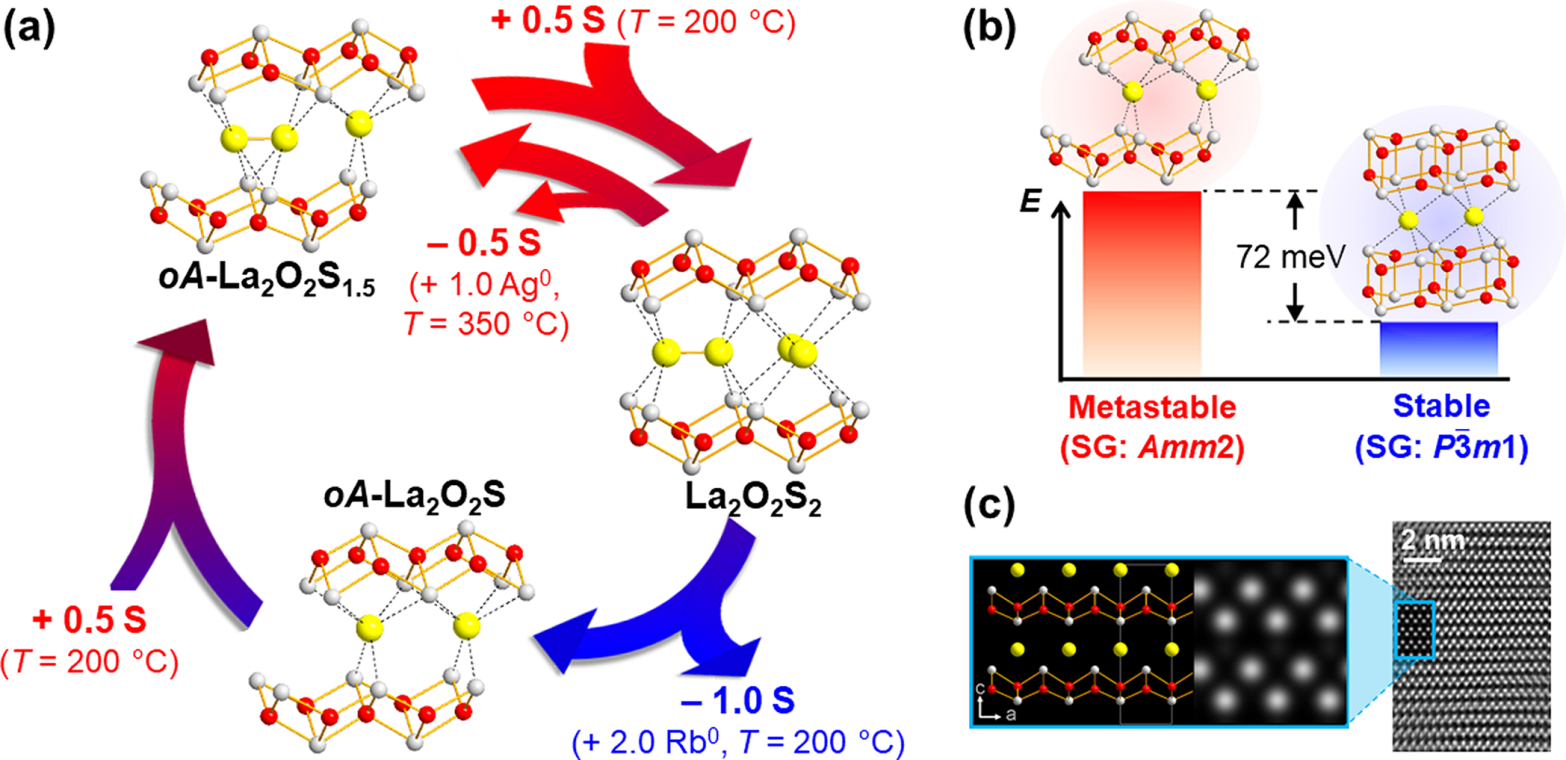
(a) Topochemical deintercalation of sulfur anions from layered oxysulfide [La_2_O_2_]^2+^[S_2_]^2–^. Reaction conditions of respective processes are noted in parentheses. (b) Two possible structures and their relative energies predicted by computational structure prediction. (c) HAADF-STEM image after the reaction with Rb^0^. It displayed good agreement with the simulated image of the *oA*-La_2_O_2_S model. Reprinted with permission under a Creative Commons Attribution 4.0 from ref (28). Copyright (2021) Springer Nature.

Reductive cleavage of chalcogen-chalcogen bonds is conducive not only to intercalation of metal cations but also to deintercalation of their chalcogen anions (Figure 1b). As described in Figure 5a, the layered oxysulfide [La_2_O_2_]^2+^[S_2_]^2–^ reacted with Cu^0^ metals to trigger its intercalation, resulting in construction of 2D [Cu_2_S_2_]^2–^ layers.^26^ On the other hand, its low-temperature (*T* = 350 °C) reaction with Ag^0^ metals did not give the corresponding quaternary phase [La_2_O_2_]^2+^[Ag_2_S_2_]^2–^, but instead produced the mixture of Ag_2_S and the novel sulfur-deficient La_2_O_2_S_1.5_ (= [La_4_O_4_]^4+^[S_2_]^2–^[S^2–^]) phase.^28^ 3D electron diffraction analysis solved the structure of this novel phase (Figure 9a); *oA*-La_2_O_2_S_1.5_ (*oA*: Pearson’s notation) crystallizes in the *Amm*2 space group, where one-fourth of the sulfur anions were removed through cleavage of some of the S–S bonds. Throughout the process, the PbO-type [La_2_O_2_]^2+^ slabs retained their structural integrity, indicating the topochemical nature of this sulfur deintercalation.

This discovery encouraged us to go further on sulfur deintercalation. La_2_O_2_S is the *x* = 1 end member of the La_2_O_2_S_2–*x*_ series and belongs to one of the most well-known oxysulfide family Ln_2_O_2_S (Ln = rare-earth elements except Sc and *Pm*). They have been serving as excellent matrices for phosphor dopants and found in various applications ranging from cathode ray tubes, laser emission/absorption, scintillators to bioimaging.^83^ This known La_2_O_2_S phase could be prepared simply by desulfurizing La_2_O_2_S_2_ under 5% H_2_/Ar flow under 350 °C,^28^ but its hexagonal structure (*hP*-La_2_O_2_S in Pearson’s notation) with the *P*3̅*m*1 space group no longer retained the quasi-tetragonal [La_2_O_2_]^2+^ slabs of La_2_O_2_S_2_ (Figure 9b). On the other hand, our computational structure prediction using the evolutionary algorithm USPEX^84^ have suggested, besides the most stable *hP*-La_2_O_2_S, another polymorph with the space group *Amm*2. This metastable polymorph, namely, *oA*-La_2_O_2_S, has the quasi-tetragonal PbO-type [La_2_O_2_]^2+^ slabs common with La_2_O_2_S_2_. To reach this hypothetical metastable phase, La_2_O_2_S_2_ was treated with Rb^0^, an alkali metal sufficiently reducing to cut all S–S bonds but too large to be inserted into the host framework.^28^ The reaction proceeded quickly at 200 °C to give the predicted *oA*-La_2_O_2_S phase, whose HAADF-STEM image clearly evidenced PbO-type [La_2_O_2_]^2+^ slabs inherited from La_2_O_2_S_2_ (Figure 9c).

As expected from their topochemical relationship, both *oA*-La_2_O_2_S_1.5_ and *oA*-La_2_O_2_S could be brought back to La_2_O_2_S_2_ by treating with sulfur at 200 °C. This reversible (de)intercalation chemistry is not limited to the lanthanum system but was recently extended to other Ln_2_O_2_S_2_ (Ln = Pr, Nd) precursors, which reacted readily with alkali metals to give the sulfur-deintercalated *oA*-Ln_2_O_2_S phases.^85^

Those are the first successful syntheses of novel metastable compounds through the low-temperature deintercalation of sulfur anions. Compared to oxygen or fluorine anions, insertion and removal of the bulky chalcogen anions are more likely to end up with nontopochemical, destructive structure transformations. However, there are several inspiring reports supporting the feasibility of sulfur (de)intercalation chemistry. One such example is the high-temperature topochemistry of Cs_2_[Ga_2_[Ch_2_]_2–*x*_Ch_2+*x*_] (Ch = S, Se; *x* = 0, 1, 2) reported by Friedrich et al.^86,87^ They studied thermal decomposition behaviors of the 1D selenogallate Cs_2_[Ga_2_[Ch_2_]_2_Ch_2_] (= CsGaCh_3_), where GaCh_4_ tetrahedra share vertexes and are further connected by anionic Ch–Ch bonds at another apex. Those 1D polychalcogenides underwent decomposition into Cs_2_[Ga_2_[Ch_2_]Ch_3_] (= Cs_2_Ga_2_Ch_5_) and Cs_2_[Ga_2_Ch_4_] (= CsGaCh_2_) at 540–680 °C (Ch = Se)^86^ and 440–540 °C (Ch = S).^87^ The products of the thermal decomposition retained the 1D chain structure of GaCh_4_ tetrahedra, but their Ch–Ch bonds were broken, and half of their chalcogen anions were deintercalated from the system. In the case of Ch = Se, the deintercalation could be reversed through the reaction with elemental Se at 500–600 °C, implying the topochemical nature of the process.

Another intriguing example can be found in nature. Lapis lazuli, or its ground form, ultramarine, is a metamorphic rock famous for its bright blue color. The blue color is known to come from the presence of [S_3_]^−^ radical anions embedded in β-cages of its zeolite LTA framework,^88^ and this sulfur-containing component can be formulated as Na_6_(Al_6_Si_6_O_24_)·2NaS_*x*_ (*x* = 2–3; note that bulk compositions of ultramarines are much more complex containing, e.g., Ca SO_4_, Cl, and OH ions). Actually, these blue [S_3_]^−^ species in β-cages could be enriched by heating with elemental sulfur^89^ or under dynamic vacuum.^90^ These observations suggested the migration of sulfur anions in the zeolite framework.

Except for the few cases described here, sulfur anions are generally reckoned to be an immobile species in solids, and their solid-state diffusion is scarcely documented. Nevertheless, Ushakova and co-workers carried out a series of attempts to evaluate diffusion constants of S^2–^ anions in solid electrolytes using Hebb-Wagner type cells^91^ and other electrochemical setups.^92^ According to their reports, several ternary sulfides ALn_2_S_4_ (A = Ca, Ba; Ln = Sm, Gd, Yb) exhibited faster S^2–^ diffusion when doped with the binary sulfides Ln_2_S_3_, and the activation energy of S^2–^ diffusion could be much lower than 1.0 eV.^91^ This possible Ch^2–/–^ diffusion was corroborated by the recent report from Lei et al.;^93^ they demonstrated electrochemical (de)intercalation of Se^2–/–^ anions in between vdW gap of 2D MoSe_2_. Those results encourages further in-depth analyses from methodological and theoretical viewpoints,^94^ as well as possible applications to electrochemical devices based on sulfur-ion shuttling,^95^ similar to fluoride-ion batteries that have attracted much recent attention.^96^

## Perspectives in Syntheses and Characterizations

Compared to the number of functional materials^3−22^ discovered by cationic redox topochemistry, there are still only a handful of examples in which novel compounds were synthesized through topochemistry involving anion–anion bond cleavage or formation (Table 1). One of the major reasons for the scarcity is the complex reaction manifolds of anionic redox topochemistry where multiple side reactions compete with each other.

**Table 1:** Metastable Compounds Discovered Using Anionic Redox Topoichemistry

product	precursor	synthesis method	redox center	reaction type	literature
Li_3_MCh_3_^a^	MCh_3_ (M = Ti, Zr, Hf, Nb; Ch = S, Se)	chemical, electrochemical	M^3+/4+^, Ch^2–/–^	metal intercalation into quasi-1D vdW system	(55−60)
Rb_0.4_ZrSe_3_	ZrSe_3_	chemical	Se^2–/–^	metal intercalation into quasi-1D vdW system	(50)
Ag_2.5_Zr_1-δ_Te_3_	ZrTe_3_	electrochemical	Zr^3+/4+^, Te^2–/–^	metal intercalation into quasi-1D vdW system	(51)
Mg_3_V_2_S_8_	VS_4_	electrochemical	V^4+/5+^, S^2–/–^	metal intercalation into 1D vdW system	(52)
metastable 2D FeS_2_^a^	Li_2_FeS_2_	chemical, electrochemical	Fe^2+/3+^, S^2–/–^	metal deintercalaion to form 2D vdW system^b^	(45−49)
Sr_2_MnO_2_Ch_2_	Sr_2_MnO_2_Cu_1.5_Ch_2_ (Ch = S, Se)	chemical	Ch^2–/–^	“zipper”-type deintercalation of metal cations	(74)
Bi_2_O_2_Ch_2_	Bi_2_O_2_Cu_2_Ch_2_ (Ch = S, Se)	chemical, electrochemical	Ch^2–/–^	“zipper”-type deintercalation of metal cations	(71)
Li[NiB]_*n*_ (*n* = 2, 3)	LiNiB	chemical	[NiB]^−^/[NiB]^−1/*n*^	“zipper”-type deintercalation of metal cations	(27)
Ln_2_O_2_S_2–*x*_ (*x* = 0.5, 1.0)^c^	Ln_2_O_2_S_2_ (Ln = La, Pr, Nd)	chemical	S^2–/–^	“zipper”-type deintercalation of chalcogen anions	(28, 85)

a Structures of those products have not been fully identified yet.

b According to the proposed structures reported in refs (48),^49^.

c La_2_O_2_S (the end member at *x* = 1.0) crystallized in the metastable polymorph with *Amm*2 space group. See Figure 9 for details.

Already in early studies during the 1970s, competition between metal intercalations (e.g., MCh_3_ + *x*A → A_*x*_MCh_3_; A = alkali metals, M = transition metals) and conversion reactions (e.g., MCh_3_ + 2*x*A → MCh_3–*x*_ + *x*A_2_Ch) have been recognized.^7,56^ To date, there is no universal strategy to control this competition, but here, we note some synthetic methods that led to successful isolation of the metal-intercalated products suppressing the competing conversion processes.

**Figure 10:**
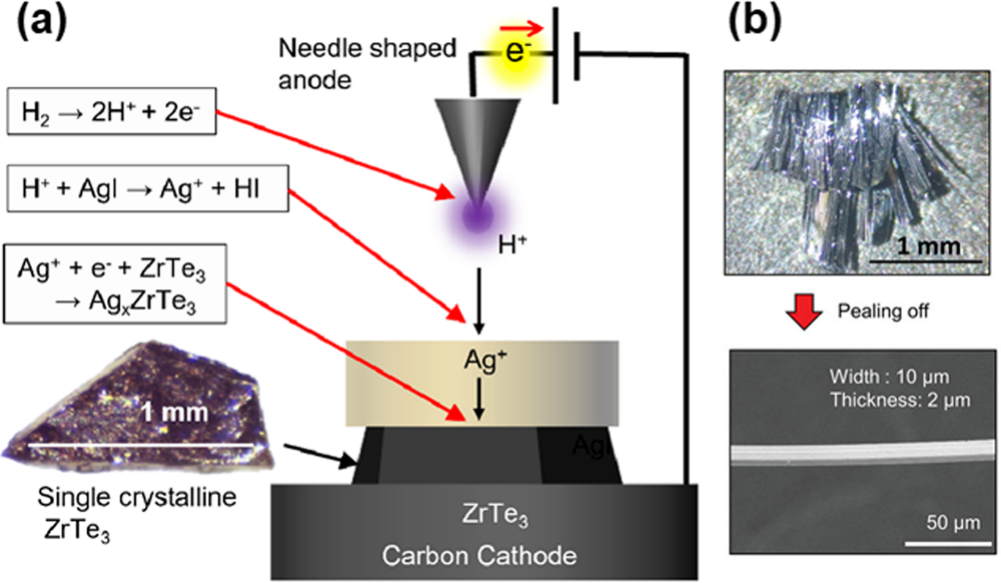
(a) Schematic illustration of proton-driven ion introduction (PDII) used for Ag^+^ intercalation into ZrTe_3_ described in Figure 4a. (b) Photographs of the as-prepared sample after Ag intercalation and the fibrous crystal peeled off from them. Reprinted with permission from ref (51). Copyright (2023) Wiley-VCH.

One viable strategy to harness the competition is electrochemical syntheses since they regulate the introduction of metal intercalants and can be used to avoid progression to conversion reactions. Such control of electrochemical reductions successfully gave new compounds via Mg intercalation into VS_4_ (Figure 4b)^52^ and Li intercalation into Bi_2_O_2_Ch_2_ (Figure 5b).^71^ While host materials in those electrochemical syntheses were in powder form and mixed with carbon compounds, Fujioka et al. prepared single crystalline Ag-intercalated ZrTe_3_ (Figure 4a).^51^ They developed the novel method for solid-state intercalation called proton-driven ion introduction^97^ (PDII; see Figure 10), where a high voltage between the needle-shaped anode and carbon cathode ionized a hydrogen atmosphere, followed by bombardment of H^+^ ions onto a AgI surface, driving Ag^+^ cations toward ZrTe_3_ at the cathode. As a consequence, ZrTe_3_ respectively received Ag^+^ from the AgI electrolyte and electrons from the cathode, producing Ag_*x*_ZrTe_3_ without destroying its single-crystalline morphology. Thanks to this, the superconducting behavior of Ag_*x*_ZrTe_3_ single crystals could be evaluated as a function of Ag content *x*. Such single-crystal-to-single-crystal (SCSC) routes^98^ may be beneficial also for suppressing conversion process given the recent *in situ* TEM study by Luo et al.;^61^ they found that Li intercalation into NbSe_3_ was favored over a competing conversion process in the large crystals while the opposite was observed with small crystals. Those results encourage further comprehensive investigations of the relationship between crystal morphology and the selectivity of the anionic redox topochemistry.

Soft-chemical processes are an alternative approach to isolating intercalation compounds. Traditionally, *n*-butyllithium was employed for chemical lithiation^7^ while I_2_, Br_2_ and NO_2_BF_4_ were used for oxidative deintercalation.^38,72^ The use of I_2_ was found effective also for anionic redox topochemistry, as exemplified by deintercalation of Li^+^ from LiFeS_2_ (Figure 3)^45^ and Cu^+^ from La_2_O_2_Cu_2_S_2_ and Bi_2_O_2_Cu_2_Ch_2_ (Ch = S, Se; Figure 5).^26,71^ Nevertheless, those oxidizing reagents sometimes led to decomposition of host materials such as Sr_2_MnO_2_Cu_1.5-x_Ch_2_.^73^ In this case, undesirable side reactions may be circumvented, as described in Figure 6, by going through reactive intermediate (e.g., Sr_2_MnO_2_Li_*x*_Ch_2_) and by employing chemoselective reagents (e.g., Difulfiram for S^2–/–^ oxidation).^74^ Such multistep syntheses through energetic intermediates are a part of routine practices in organic chemistry, and are increasingly recognized as an important strategy also in solid-state chemistry (see, e.g., refs (99) and (100) where insertion of H^+^ or H^–^ ions rendered host oxide frameworks less stable, enabling more challenging topochemical transformations).

This section highlights a few soft-chemical routes successfully used in the reported cases (see Table 1). However, many other synthesis methods could potentially be applied to control anionic redox topochemistry. For instance, metathesis reactions have often been utilized in syntheses of polychalcogenides.^101^ These reactions can therefore serve as a driving force for introducing chalcogen-chalcogen bonds in a topochemical manner. Indeed, metathesis reactions have successfully introduced chalcogen anions in between the host cationic slabs, resulting in [Hf_2_N_2_]^2+^[S^2–^] from [Hf_2_N_2_]^2+^[Cl^–^]_2_ and Ti_3_C_2_Ch (Ch = S, Te) from Ti_3_C_2_Br_2_ MXene.^102,103^ While these examples did not involve anionic redox, this alternative driving force may provide an opportunity to expand the applicability of chalcogen (de)intercalation beyond the *oA*-Ln_2_O_2_S_2-x_ system described in Figure 9.

**Figure 11:**
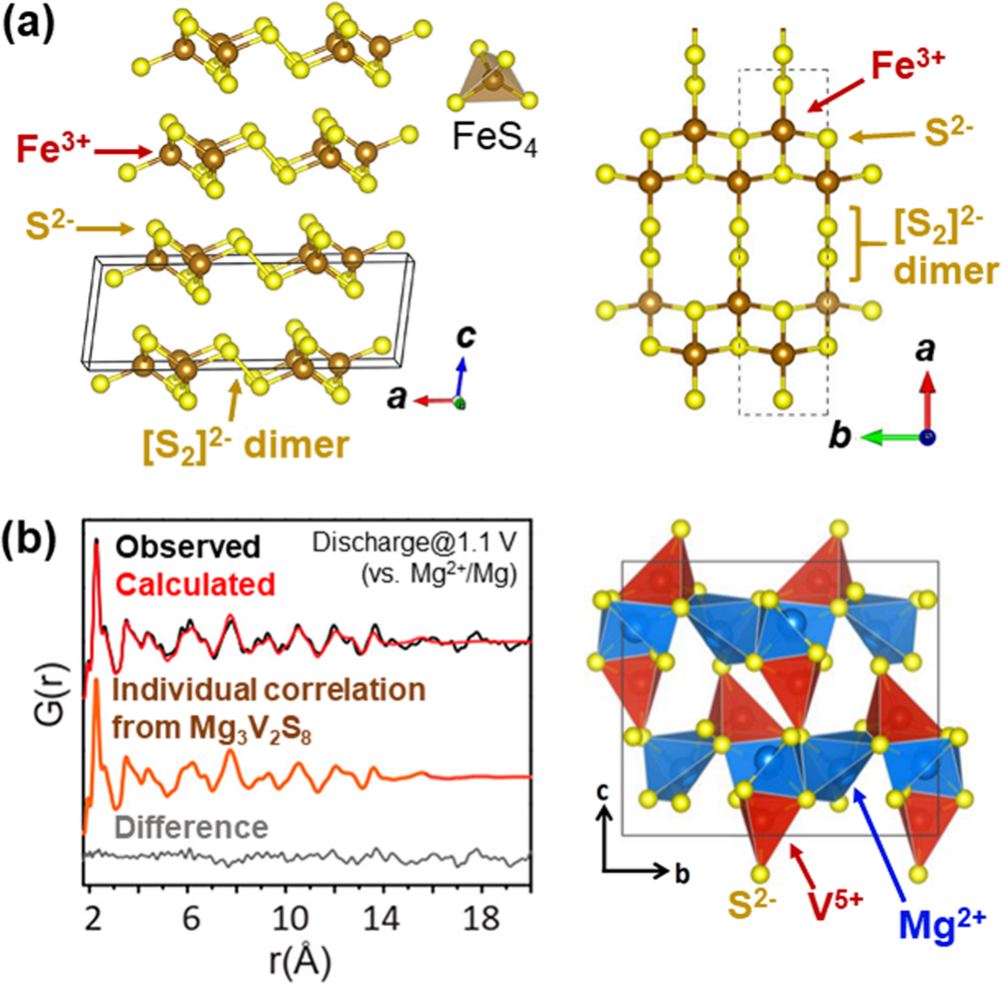
Uses of computational structure prediction for structure characterizations. (a) The structure of Fe^3+^S^2–^[[S_2_]^2–^]_1/2_ (i.e., metastable FeS_2_) predicted by the evolutionary algorithm USPEX. See Figure 3 for its electrochemical behavior. (b) X-ray pair distribution function data after electrochemical Mg intercalation into VS_4_ using the USPEX-predicted Mg_3_V_2_S_8_ structure. The right panel shows the structure after refinement. See also Figure 4b. Reprinted from refs (49) and (52). Copyright (2020) American Chemical Society.

Structure characterization is another major challenge in anionic redox topochemistry. Generally speaking, anion–anion bond formation and cleavage resulted in relatively poor crystallinity of the topochemical products. Two seminal examples, Li-intercalated Li_*x*_MS_3_ (M = Ti, Zr, Hf; *x* ∼ 3) and the metastable FeS_2_ from Li deintercalation of Li_*x*_FeS_2_ (see Figure 3), were both reported over 40 years ago,^45,55^ but their structures could not be solved by conventional diffraction methods. Recently, Hansen et al. revisited this electrochemical Li deintercalation from Li_*x*_FeS_2_ employing *in operando* XRD, *ex-situ* XANES and EXAFS analyses.^48^ Their high-quality data confirmed that Li deintercalation oxidized both Fe^2+^ and S^2–^, but the FeS_4_ tetrahedra remained undistorted, confirming the suggestions from 1980s Mössbauer and EXAFS studies.^46^ Also from the theoretical side, Wang et al. carried out computational structure prediction^49^ using the evolutionary algorithm USPEX.^84^ They found the series of structures in FeS_2_ compositional space including Pyrite, Marcasite and the new metastable structure with *C*2/*m* space group (Figure 11a). This 2D vdW structure was composed of 1D [Fe_2_S_4_] ribbons made of edge-sharing FeS_4_ tetrahedra and bridged by S–S bonds. Its calculated band structure confirmed high-spin Fe^3+^ cations in tetrahedral coordination, coexisting with [S_2_]^2–^ and S^2–^ species. This observation was also consistent with early experimental studies.^45,46^

Obviously, those computational structure predictions become extremely useful when they are combined with local probe techniques. One good example was characterization of Mg_3_V_2_S_8_ prepared by Mg intercalation into VS_4_ (Figure 4b).^52^ Although the chemical nature of Mg_3_V_2_S_8_ could be well characterized by their ^51^V NMR, XPS, and XANES analyses, its XRD did not show any diffraction arising from this Mg-intercalated phase. The hypothetical structure model was first acquired using USPEX, which was then subject to refinement against the pair distribution function data (PDF; see Figure 11b). The USPEX-predicted structure showed good agreement with the experimental X-ray pattern and revealed its structural evolution during electrochemical cycling.

**Figure 12:**
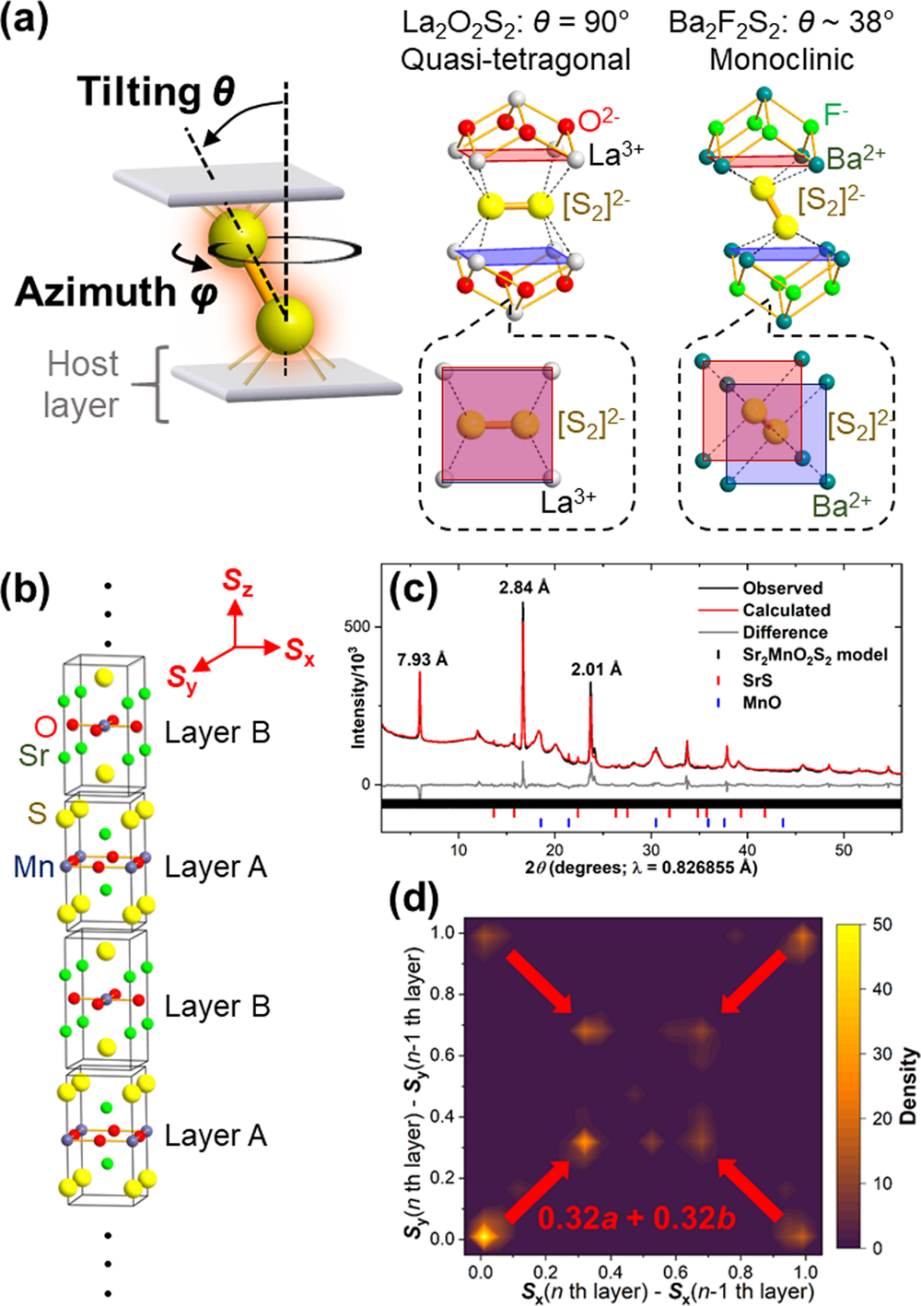
(a) Rotational degree of freedom of [Ch_2_]^2–^ dimers and its effects on stacking of host cationic layers in the cases of La_2_O_2_S_2_ and Ba_2_F_2_S_2_. (b) A hundred-layer supercell used for modeling disordered stacking in Sr_2_MnO_2_Ch_2_ (see Figure 6) and (c) its Rietveld fit against the synchrotron XRD pattern. (d) 2D histogram showing refined in-plane stacking vectors (*S*_*x*_, *S*_*y*_) at respective layers relative to adjacent ones. Panels (b)–(d) were reprinted with permission under a Creative Commons Attribution 4.0 from ref (74). Copyright (2023) Springer Nature.

Besides those generic crystallinity issues, stacking disorder has been a long-standing challenge in the structure characterization of intercalation compounds. 2D vdW systems and intercalation compounds (Figure 1a) often exhibit irregular stacking sequences. This causes severe *hkl*-dependent broadening of diffraction peaks, which must be modeled using large multilayered supercells.^104,105^ In that sense, “zipper”-type architecture of polychalcogenides (Figure 1b) seems less susceptible to such stacking disorder, owing to their 3D networks interconnected by covalent anion–anion bonds. Contrary to such expectation, severe *hkl*-dependent peak broadening was observed also in the XRD of Sr_2_MnO_2_Ch_2_ (Figure 12c), a layered polychalcogenide “zipped up” by multistep Cu deintercalation (see Figure 6). Stacking in layered polychalcogenides has a certain extent of flexibility arising from an orientational degree of freedom of chalcogen dimers (Figure 12a). For example, [S_2_]^2–^ dimers in La_2_O_2_S_2_ completely lie in the its basal plane while those in Ba_2_F_2_S_2_ tilt from the stacking axis by θ ∼ 38°, causing a different stacking sequence of the host PbO-type layers. Additionally, those [S_2_]^2–^ dimers may be subject to in-plane axial rotation by, e.g., φ = ± 90°, which does not change local environment around the dimer, but introduce irregularity in their periodic stacking sequences (see ref (68) for the case study in La_2_O_2_S_2_).

The XRD pattern in Figure 12c can be understood as an extreme case of such a stacking disorder of layered polychalcogenides. HAADF-STEM of the Sr_2_MnO_2_Ch_2_ sample evidenced an irregular stacking sequence of their perovskite-type slabs, most likely arising from various orientation of chalcogen dimers.^74^ In addition, ^7^Li NMR revealed that residual Li/Cu intercalants also formed stacking faults. This complex stacking disorder was modeled by a large supercell approach in which one hundred Sr_2_MnO_2_Ch_2_ layers were allowed to move individually relative to one another during the refinement (Figure 12b). This free-parameter approach similar to the one reported by Metz et al.^106^ has successfully identified the most frequent stacking vectors (*S*_*x*_, *S*_*y*_) in their disordered stacking (Figure 12d). Such characterizations of stacking vectors provide ideas about possible local structures of disordered low-dimensional polychalcogenides. This conjectures may be further corroborated by other local probe analyses and computational structure predictions, ultimately enabling identifications of detailed structure models.

## Conclusion

This Perspective presents a brief overview of anionic redox topochemistry as a tool to design novel solid-state materials. The basic concept of anionic redox topochemistry was established more than 30 years ago,^34,40,41^ and several polychalcogenides have been examined as host frameworks.^45,55,56^ These seminal studies were followed by extensive investigations aiming at secondary battery applications because anionic redox can be a promising way to boost energy density of cathode materials.^36,42−44^ On the other hand, unique structure transformations involving anion–anion bond cleavages or formations have never been elucidated until a surge of such studies in the last 5 years.^26−28,50−52,70,71,74^ This is in stark contrast to cationic redox topochemistry, where its structural chemistry and its uses to design novel electronic or magnetic materials^7^ were examined in parallel with its application to secondary batteries.^13^ This absence of materials design by anionic redox topochemistry can be ascribed to poor crystallinity of the products and its complex reaction manifolds where intercalation, conversion, and many other nontopochemical processes compete with each other. Recent advancements in syntheses and characterizations are resolving these issues.

Despite its challenging nature, anionic redox topochemistry enables unique types of structure transformations. One such process highlighted in this Perspective was “zipper”-type topochemistry (Figure 1b). Both processes, i.e., in situ 2D layer construction and topochemical conversion, clearly highlight the promise of anionic redox topochemistry. Now the next challenge will be to design functional materials with, e.g., promising electronic, magnetic, optical, and catalytic properties. As exemplified throughout the Perspective, such materials design will be achieved most likely through close collaborations between diverse experts across synthetic and computational chemistry, crystallography, spectroscopy, and condensed matter physics.

## Data Availability

The data underlying this study are available in the published article.

## Abbreviations

vdW: van der Waals
HAADF-STEM: High-angle annular dark-field scanning transmission electron microscopy
XANES: X-ray absorption near-edge structure
XPS: X-ray photoelectron spectroscopy
USPEX: Universal Structure Predictor: Evolutionary Xtallography
